# Association of Female Reproductive Factors With Incidence of Fracture Among Postmenopausal Women in Korea

**DOI:** 10.1001/jamanetworkopen.2020.30405

**Published:** 2021-01-06

**Authors:** Jung Eun Yoo, Dong Wook Shin, Kyungdo Han, Dahye Kim, Ji Won Yoon, Dong-Yun Lee

**Affiliations:** 1 Healthcare System Gangnam Center, Department of Family Medicine, Seoul National University Hospital, Seoul, Republic of Korea; 2 Department of Family Medicine and Supportive Care Center, Samsung Medical Center, Sungkyunkwan University School of Medicine, Seoul, Republic of Korea; 3 Department of Clinical Research Design and Evaluation, Samsung Advanced Institute for Health Science & Technology (SAIHST), Sungkyunkwan University, Seoul, Republic of Korea; 4 Department of Statistics and Actuarial Science, Soongsil University, Seoul, Republic of Korea; 5 Department of Medical Statistics, The Catholic University of Korea, Seoul, Republic of Korea; 6 Healthcare System Gangnam Center, Department of Internal Medicine, Seoul National University Hospital, Seoul, Republic of Korea; 7 Department of Obstetrics and Gynecology, Samsung Medical Center, Sungkyunkwan University School of Medicine, Seoul, Republic of Korea

## Abstract

**Importance:**

Although estrogen level is positively associated with bone mineral density, there are limited data on the risk of fractures after menopause.

**Objective:**

To investigate whether female reproductive factors are associated with fractures among postmenopausal women.

**Design, Setting, and Participants:**

This population-based retrospective cohort study used data from the Korean National Health Insurance Service database on 1 272 115 postmenopausal women without previous fracture who underwent both cardiovascular and breast and/or cervical cancer screening from January 1 to December 31, 2009. Outcome data were obtained through December 31, 2018.

**Exposures:**

Information was obtained about reproductive factors (age at menarche, age at menopause, parity, breastfeeding, and exogenous hormone use) by self-administered questionnaire.

**Main Outcomes and Measures:**

Incidence of any fractures and site-specific fractures (vertebral, hip, and others).

**Results:**

Among the 1 272 115 participants, mean (SD) age was 61.0 (8.1) years. Compared with earlier age at menarche (≤12 years), later age at menarche (≥17 years) was associated with a higher risk of any fracture (adjusted hazard ratio [aHR], 1.24; 95% CI, 1.17-1.31) and vertebral fracture (aHR, 1.42; 95% CI, 1.28-1.58). Compared with earlier age at menopause (<40 years), later age at menopause (≥55 years) was associated with a lower risk of any fracture (aHR, 0.89; 95% CI, 0.86-0.93), vertebral fracture (aHR, 0.77; 95% CI, 0.73-0.81), and hip fracture (aHR, 0.88; 95% CI, 0.78-1.00). Longer reproductive span (≥40 years) was associated with lower risk of fractures compared with shorter reproductive span (<30 years) (any fracture: aHR, 0.86; 95% CI, 0.84-0.88; vertebral fracture: aHR, 0.73; 95% CI, 0.71-0.76; and hip fracture: aHR, 0.87; 95% CI, 0.80-0.95). Parous women had a lower risk of any fracture than nulliparous women (aHR, 0.96; 95% CI, 0.92-0.99). Although breastfeeding for 12 months or longer was associated with a higher risk of any fractures (aHR, 1.05; 95% CI, 1.03-1.08) and vertebral fractures (aHR, 1.22; 95% CI, 1.17-1.27), it was associated with a lower risk of hip fracture (aHR, 0.84; 95% CI, 0.76-0.93). Hormone therapy for 5 years or longer was associated with lower risk of any factures (aHR, 0.85; 95% CI, 0.83-0.88), while use of oral contraceptives for 1 year or longer was associated with a higher risk of any fractures (aHR, 1.03; 95% CI, 1.01-1.05).

**Conclusions and Relevance:**

The findings of this cohort study suggest that female reproductive factors are independent risk factors for fracture, with a higher risk associated with shorter lifetime endogenous estrogen exposure. Interventions to reduce fracture risk may be needed for women at high risk, including those without osteoporosis.

## Key Points

**Question:**

Are female reproductive factors associated with bone health in postmenopausal women?

**Findings:**

In this population-based cohort study of 1 272 115 postmenopausal Korean women, later menarche, earlier menopause, and shorter reproductive span were each independently associated with increased risk of fracture.

**Meaning:**

This study’s findings suggest that female reproductive factors are independent risk factors for fracture incidence, with a higher risk associated with shorter lifetime endogenous estrogen exposure.

## Teaser

This cohort study uses data from the Korean National Health Insurance System database to investigate whether female reproductive factors are associated with fractures among postmenopausal women in Korea.

## Introduction

Osteoporotic fractures are an important global health issue in aging societies. The combined lifetime risk for any type of fracture that receives clinical attention is around 40%, which is equivalent to the risk for cardiovascular disease.^1^ In particular, postmenopausal women disproportionately experience osteoporosis and its sequelae, such as fractures.^2^

Estrogen levels are positively associated with bone mineral density (BMD) and have been suggested to have protective effects against osteoporotic fractures. For example, epidemiologic studies have shown that late age at menarche is associated with a risk of reduced BMD^3^ and subsequent osteoporosis or osteoporotic fractures.^3,4,5^ In addition, earlier menopause^4,6,7,8,9,10^ and shorter reproductive span^3,4,5,8,11^ have all been suggested to be risk factors for osteoporotic fractures. However, some reported fracture data were based on questionnaires or interviews and thus may have been subject to recall bias.^4,5,6,7,10^ In addition, limitations of previous studies included assessment of only certain types of fractures (eg, hip fractures^4,8,10^ or vertebral fractures^5^) and failure to adjust for comorbidities that may act as potential confounders, such as diabetes or cancer.^4,5,9,10^ Although there is wide variation in BMD and fracture incidence across racial and ethnic groups,^12^ epidemiologic information regarding osteoporotic fractures in Asian women and its determinants is scarce, comprising 1 Japanese study involving 250 self-reported vertebral fractures among 43 652 women^5^ and 1 Chinese study with 1327 incident hip fractures among 125 336 women.^8^ Furthermore, BMD levels change during pregnancy and lactation, but the long-term associations of these events with postmenopausal BMD or fracture are controversial, with protective,^13,14,15^ negative,^16,17,18^ and null^4,5^ findings.

Hormone therapy (HT) reduces postmenopausal bone loss and decreases the incidence of all osteoporosis-related fractures^19^ even in women not at high risk of fractures.^20^ In a meta-analysis of randomized clinical trials, HT was associated with a 26% overall reduction in total fractures, a 37% reduction in vertebral fractures, and a 28% reduction in hip fractures.^21^ However, because all published randomized clinical trials have been conducted in Western countries, the associations of HT with the risk of fractures in Asian women is unclear. The associations of exogenous estrogen exposure from oral contraceptives (OCs) with fracture risk also remain inconclusive.^22^ Use of OCs was reported to be protective against low BMD in several studies,^23,24,25^ but null^26,27,28^ or even negative^29^ associations have also been reported. It is also unclear whether the associations of OC use with BMD during reproductive years continue into postmenopausal years when bone loss accelerates and if such changes in BMD alter the risk of fracture later in life.^30,31^ Therefore, in this retrospective cohort study of a large population of women identified from a population-based database, we investigated the associations between female reproductive factors and incident fractures.

## Methods

### Data Source and Study Setting

We analyzed data obtained from the Korean National Health Insurance Service (NHIS), which contains sociodemographic information. To reimburse medical providers, the NHIS collects all information about use of medical facilities as well as records of medical procedures and prescriptions with *International Statistical Classification of Diseases and Related Health Problems, Tenth Revision* (*ICD-10*) codes. In addition, the NHIS recommends free biennial cardiovascular health screening for all Koreans 40 years or older and all employees regardless of age, as well as annual screening for workers in jobs requiring physical labor, enabling the NHIS to collect data from health check-ups (self-administered questionnaire on health behavior, anthropometric measurements, and laboratory test results).^32,33^ This study was designed and conducted according to the Strengthening the Reporting of Observational Studies in Epidemiology (STROBE) reporting guideline.^34^ This study received institutional review board approval from Samsung Medical Center. Informed consent was waived because the data are public and anonymized under confidentiality guidelines.

The National Cancer Screening Program was implemented as part of the Korean National Cancer Control Plan and involves cancer screening of all individuals exceeding cancer-specific target ages.^35^ For instance, all Korean women older than 40 years are screened for breast cancer biennially and are required to fill out a self-administered questionnaire on reproductive factors.^35^

### Study Population

Among 3 109 506 women 40 years or older who underwent both cardiovascular and breast and/or cervical cancer screening from January 1 to December 31, 2009, we identified 1 939 690 eligible postmenopausal women. We first excluded individuals who reported undergoing a hysterectomy procedure in general (n = 215 083), as most did not know whether they had a simultaneous oophorectomy. We also excluded people with any fracture history (n = 111 764) or registered disability status (n = 99 861) before the health screening date and those with at least 1 missing variable (n = 216 913). Last, those who had any fracture (n = 20 760) or died (n = 3194) within 1 year after the health screening date were excluded. A total of 1 272 115 individuals were included in the final analyses (eFigure in the Supplement). The cohort was followed up from baseline (health screening date) to the date of incident fracture, censoring (eg, death), or end of the study period (December 31, 2018), whichever came first.

### Exposure and Study Outcome

Information about age at menarche, age at menopause, parity, total lifetime breastfeeding history, and use of HT and/or OCs was collected by self-administered questionnaire (eMethods in the Supplement). The primary end point was newly diagnosed fractures during the follow-up period. We included fracture sites considered to be the most associated with osteoporosis.^36^ Fractures were defined by *ICD-10* codes as vertebral, hip, or other fractures. Other fractures included clavicle, upper arm, wrist, and ankle (eMethods in the Supplement).^37,38^

### Statistical Analysis

Continuous variables are presented as mean (SD) values, and categorical variables are presented as numbers and percentages. Incidence rates of fractures were calculated by dividing the number of incident cases by 1000 person-years. Three multivariable Cox proportional hazards regression models were evaluated. Model 1 was the crude model. Model 2 included age, age at menarche, age at menopause, parity, duration of breastfeeding, duration of HT, duration of OC use, lifestyle risk factors (alcohol consumption, smoking, and regular exercise), income, body mass index, and comorbidities (hypertension, diabetes, dyslipidemia, and cancer). Model 3 included reproductive span instead of age at menarche and the menopause variables in model 2. Model 3 also adjusted for other reproductive factors (parity, duration of breastfeeding, duration of HT, and duration of OC use) and covariates (age, lifestyle factors, income, body mass index, and comorbidities) included in model 2. Detailed information on the variables included in multivariable models is provided in the eMethods in the Supplement.

Statistical analyses were performed using SAS, version 9.4 (SAS Institute Inc). Statistical significance was defined as 2-sided *P* < .05.

## Results

### Baseline Characteristics of the Study Population

Characteristics of the 1 272 115 study participants are presented in the Table. The mean (SD) age of the total population was 61.0 (8.1) years. The overall mean (SD) age at menarche was 16.4 (1.8) years and at menopause was 50.1 (4.0) years. The mean (SD) reproductive span was 33.6 (4.4) years. Compared with women with no fractures (n = 1 082 232), women with incident fracture (n = 189 883) were likely to be older (mean [SD] age, 63.7 [8.1] vs 60.5 [8.1] years), multiparous (92.8% vs 91.0%), have later menarche (mean [SD] age at menarche, 16.7 [1.8] vs 16.4 [1.8] years), earlier menopause (mean [SD] age at menopause, 49.9 [4.2] vs 50.1 [4.0] years), a shorter reproductive span (mean [SD], 33.3 [4.6] vs 33.7 [4.3] years), and a duration of breastfeeding of 12 months or longer (74.0% vs 67.8%) and to have never used HT (82.6% vs 80.1%) or OC (80.0% vs 79.8%).

**Table.** Baseline Characteristics of the Study Participants

Characteristic	No. (%)			*P* value
	Total (N = 1 272 115)	No fractures (n = 1 082 232)	Fractures (n = 189 883)	
Age, mean (SD), y	61.0 (8.1)	60.5 (8.1)	63.7 (8.1)	<.001
Income, quartile				
Q1 (lowest)	291 625 (22.9)	249 802 (23.1)	41 823 (22.0)	<.001
Q2	238 175 (18.7)	204 466 (18.9)	33 709 (17.8)	
Q3	314 688 (24.7)	267 733 (24.7)	46 955 (24.7)	
Q4 (highest)	427 627 (33.6)	360 231 (33.3)	67 396 (35.5)	
Smoking status				
Never	1 224 497 (96.3)	1 041 677 (96.3)	182 820 (96.3)	<.001
Ex-smoker	13 511 (1.1)	11 652 (1.1)	1859 (1.0)	
Current smoker	34 107 (2.7)	28 903 (2.7)	5204 (2.7)	
Alcohol consumption				
None	1 110 358 (87.3)	942 633 (87.1)	167 725 (88.3)	<.001
Mild	155 185 (12.2)	134 031 (12.4)	21 154 (11.1)	
Heavy	6572 (0.5)	5568 (0.5)	1004 (0.5)	
Regular exercise	237 484 (18.7)	204 195 (18.9)	33 289 (17.5)	<.001
BMI, mean (SD)	24.2 (3.1)	24.2 (3.1)	24.2 (3.1)	.30
<18.5	26 156 (2.1)	21 883 (2.0)	4273 (2.3)	<.001
18.5 to <23	439 792 (34.6)	375 698 (34.7)	64 094 (33.8)	
23 to <25	338 511 (26.6)	287 749 (26.6)	50 762 (26.7)	
25 to <30	415 412 (32.7)	352 228 (32.6)	63 184 (33.3)	
≥30	52 244 (4.1)	44 674 (4.1)	7570 (4.0)	
Blood pressure, mean (SD), mm Hg				
Systolic	125.4 (16.1)	125.2 (16.1)	126.5 (16.2)	<.001
Diastolic	76.8 (10.2)	76.8 (10.2)	77.1 (10.1)	<.001
Fasting glucose level, mean (SD), mg/dL	99.6 (24.0)	99.4 (23.8)	100.2 (25.1)	<.001
Total cholesterol level, mean (SD), mg/dL	208.3 (43.8)	208.5 (43.4)	207.4 (45.5)	<.001
Comorbidities				
Hypertension	530 378 (41.7)	443 850 (41.0)	86 528 (45.6)	<.001
Diabetes	159 996 (12.6)	132 923 (12.3)	27 073 (14.3)	<.001
Dyslipidemia	432 129 (34.0)	366 702 (33.9)	65 427 (34.5)	<.001
Cancer	33 882 (2.7)	29 205 (2.7)	4677 (2.5)	<.001
Age at menarche, mean (SD), y	16.4 (1.8)	16.4 (1.8)	16.7 (1.8)	<.001
≤12	12 580 (1.0)	12 580 (1.0)	11 295 (1.0)	<.001
13-14	159 722 (12.6)	159 722 (12.6)	140 472 (13.0)	
15-16	500 445 (39.3)	500 445 (39.4)	430 429 (39.8)	
≥17	599 368 (47.1)	599 368 (47.1)	500 036 (46.2)	
Age at menopause, mean (SD), y	50.1 (4.0)	50.1 (4.0)	49.9 (4.2)	<.001
<40	21 101 (1.7)	17 353 (1.6)	3748 (2.0)	
40-44	71 142 (5.6)	59 281 (5.5)	11 861 (6.3)	<.001
45-49	345 678 (27.2)	294 429 (27.2)	51 249 (27.0)	
50-54	699 018 (55.0)	597 401 (55.2)	101 617 (53.5)	
≥55	135 176 (10.6)	113 768 (10.5)	21 408 (11.3)	
Reproductive span, mean (SD), y	33.6 (4.4)	33.7 (4.3)	33.3 (4.6)	<.001
<30	170 474 (13.4)	140 756 (13.0)	29 718 (15.7)	<.001
30-34	529 589 (41.6)	448 793 (41.5)	80 796 (42.6)	
35-39	490 384 (38.6)	423 003 (39.1)	67 381 (35.5)	
≥40	81 668 (6.4)	69 680 (6.4)	11 988 (6.3)	
Parity				
Nulliparity	32 006 (2.5)	27 764 (2.6)	4242 (2.2)	<.001
1	79 411 (6.2)	69 965 (6.5)	9446 (5.0)	
≥2	1 160 698 (91.2)	984 503 (91.0)	176 195 (92.8)	
Duration of breastfeeding, mo				
Never	86 477 (6.8)	76 028 (7.0)	10 449 (5.5)	<.001
<6	86 290 (6.8)	76 446 (7.1)	9844 (5.2)	
6-11	225 607 (17.7)	196 465 (18.2)	29 142 (15.4)	
≥12	873 741 (68.7)	733 293 (67.8)	140 448 (74.0)	
Duration of HT, y				
Never	1 023 519 (80.5)	866 595 (80.1)	156 924 (82.6)	<.001
<2	118 146 (9.3)	102 795 (9.5)	15 351 (8.1)	
2-4	48 812 (3.8)	42 699 (4.0)	6113 (3.2)	
≥5	37 581 (3.0)	32 858 (3.0)	4723 (2.5)	
Unknown	44 057 (3.5)	37 285 (3.5)	6772 (3.6)	
Duration of OC use, y				
Never	1 015 265 (79.8)	863 407 (79.8)	151 858 (80.0)	<.001
<1	116 384 (9.2)	99 781 (9.2)	16 603 (8.7)	
≥1	77 581 (6.1)	65 718 (6.1)	11 863 (6.3)	
Unknown	62 885 (4.9)	53 326 (4.9)	9559 (5.0)	

Abbreviations: BMI, body mass index (calculated as weight in kilograms divided by height in meters squared); HT, hormone therapy; OC, oral contraceptive.

SI conversion factors: To convert cholesterol to millimoles per liter, multiply by 0.0259; glucose to millimoles per liter, multiply by 0.0555.

### Association of Reproductive Factors With Incidence of Fractures

The median follow-up duration was 8.3 years (interquartile range, 8.0-8.6 years). There were 189 883 new cases of any fractures (14.9%), which consisted of vertebral fractures (n = 72 732), hip fractures (n = 11 153), and others (n = 106 895).

#### Menstrual History

**Figure 1.**
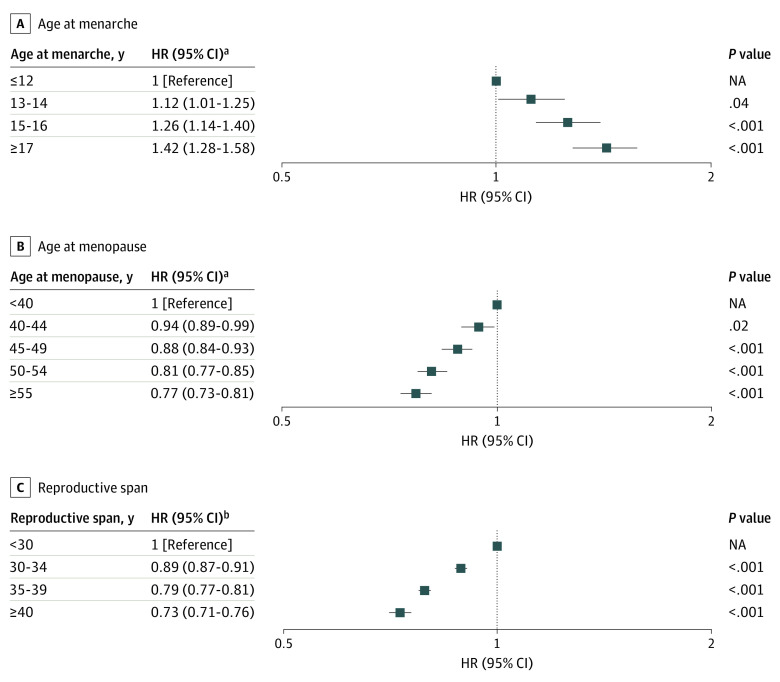
Hazard Ratios (HRs) for Vertebral Fractures According to Menstrual History NA indicates not applicable. ^a^Model 2: the full model included age, age at menarche, age at menopause, parity, duration of breastfeeding, duration of hormone therapy, duration of oral contraceptive use, alcohol consumption, smoking, regular exercise, income, body mass index, hypertension, diabetes, dyslipidemia, and cancer. ^b^Model 3: the full model included reproductive span instead of age at menarche and the menopause variables in model 2.

**Figure 2.**
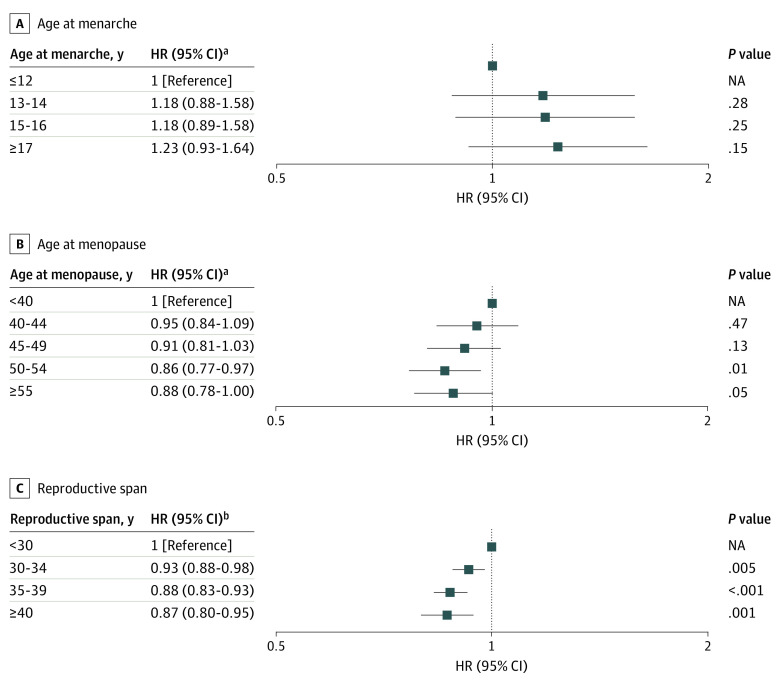
Hazard Ratios (HRs) for Hip Fractures According to Menstrual History NA indicates not applicable. ^a^Model 2: the full model included age, age at menarche, age at menopause, parity, duration of breastfeeding, duration of hormone therapy, duration of oral contraceptive use, alcohol consumption, smoking, regular exercise, income, body mass index, hypertension, diabetes, dyslipidemia, and cancer. ^b^Model 3: the full model included reproductive span instead of age at menarche and the menopause variables in model 2.

In model 2, compared with women who experienced menarche at age 12 years or younger, age at menarche showed a significant dose-response association with any fracture (age, 13-14 years; adjusted hazard ratio [aHR], 1.10; 95% CI, 1.04-1.16; 15-16 years: aHR, 1.16; 95% CI, 1.10-1.23; and ≥17 years: aHR, 1.24; 95% CI, 1.17-1.31) (eTable 1 in the Supplement). However, compared with women who experienced menopause before age 40 years, the risk of any fracture decreased with age at menopause (age, 40-44 years: aHR, 0.97; 95% CI, 0.93-1.01; 45-49 years: aHR, 0.94; 95% CI, 0.91-0.97; 50-54 years: aHR, 0.90; 95% CI, 0.88-0.93; and ≥55 years: aHR, 0.89; 95% CI, 0.86-0.93). Furthermore, in model 3, when compared with a reproductive span of less than 30 years, the risk of any fracture also decreased with reproductive span (length of span, 30-34 years: aHR, 0.94; 95% CI, 0.93-0.95; 35-39 years: aHR, 0.89; 95% CI, 0.88-0.90; and ≥40 years: aHR, 0.86; 95% CI, 0.84-0.88). Similar patterns were noted for vertebral fractures (age of menarche, ≥17 years: aHR, 1.42; 95% CI, 1.28-1.58; age at menopause, ≥55 years: aHR, 0.77; 95% CI, 0.73-0.81; and reproductive span, ≥40 years: aHR, 0.73; 95% CI, 0.71-0.76) and hip fractures (age at menopause, ≥55 years: aHR, 0.88; 95% CI, 0.78-1.00; reproductive span, ≥40 years: aHR, 0.87; 95% CI, 0.80-0.95) (eTables 2 and 3 in the Supplement, Figure 1, and Figure 2). Other fracture risk was associated only with age at menarche (eTable 4 in the Supplement).

#### Parity and Breastfeeding

**Figure 3.**
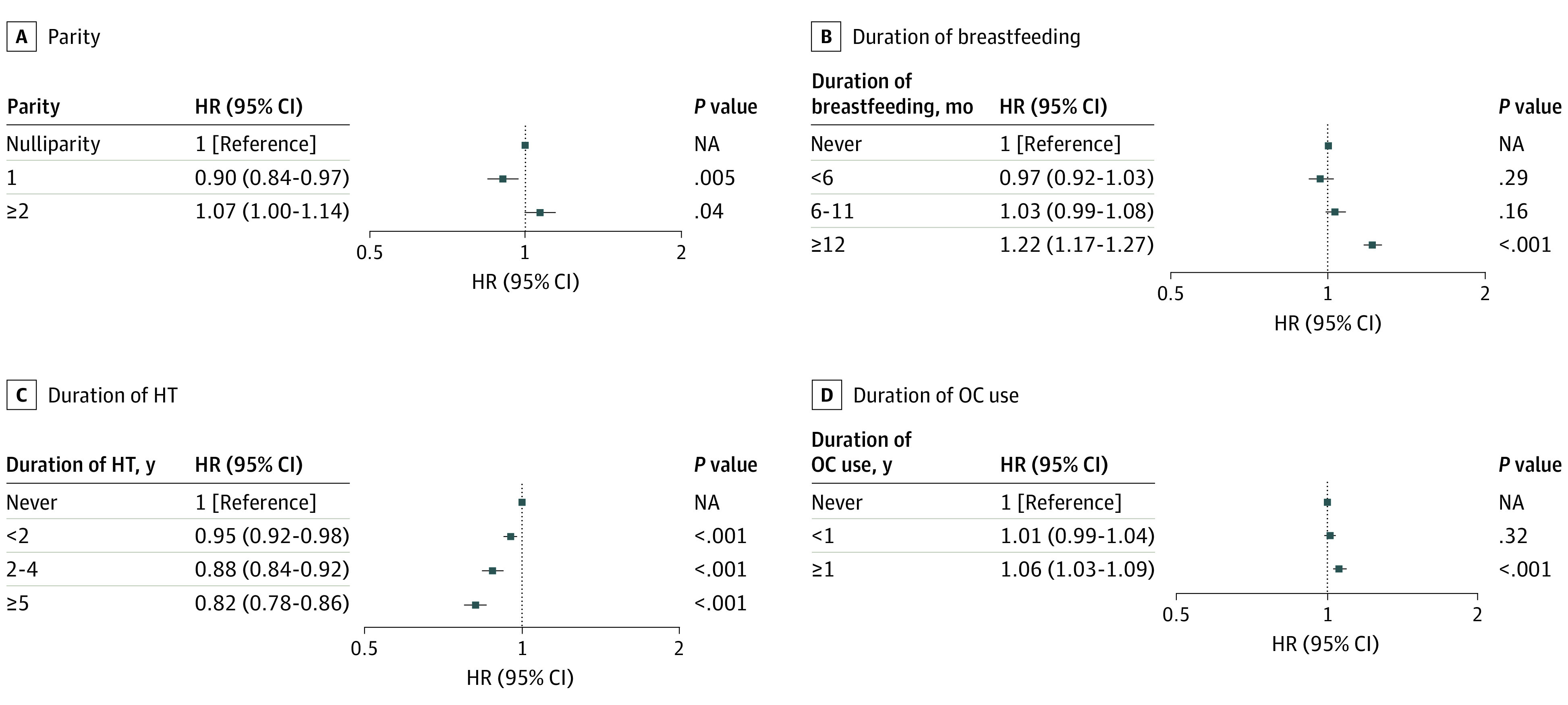
Hazard Ratios (HRs) for Vertebral Fractures According to Parity, Breastfeeding, and Exogenous Hormone Use The full model (model 2) included age, age at menarche, age at menopause, parity, duration of breastfeeding, duration of hormone therapy (HT), duration of oral contraceptive (OC) use, alcohol consumption, smoking, regular exercise, income, body mass index, hypertension, diabetes, dyslipidemia, and cancer. NA indicates not applicable.

**Figure 4.**
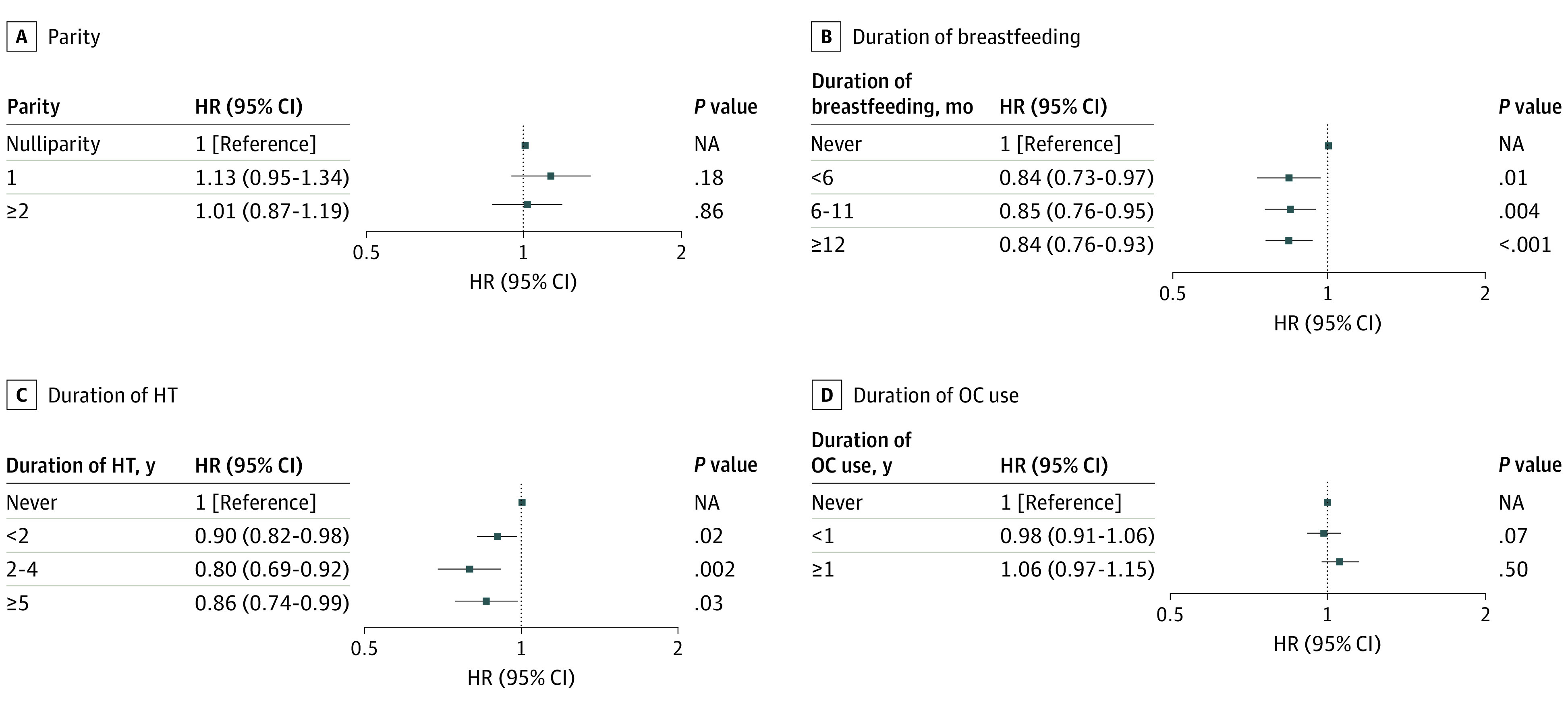
Hazard Ratios (HRs) for Hip Fractures According to Parity, Breastfeeding, and Exogenous Hormone Use The full model (model 2) included age, age at menarche, age at menopause, parity, duration of breastfeeding, duration of hormone therapy (HT), duration of oral contraceptive (OC) use, alcohol consumption, smoking, regular exercise, income, body mass index, hypertension, diabetes, dyslipidemia, and cancer. NA indicates not applicable.

In model 2, primiparous women were found to have a lower fracture risk (any fracture: aHR, 0.96; 95% CI, 0.92-0.99; vertebral fracture: aHR, 0.90; 95% CI, 0.84-0.97) than nulliparous women, whereas there was no significant association between fracture risk and multiparity (eTables 1 and 2 in the Supplement and Figure 3). There was no association between parity and hip fracture (eTable 3 in the Supplement and Figure 4). Other fracture risk was negatively associated with parity (primiparous women: aHR, 0.94; 95% CI, 0.89-0.98; multiparous women: aHR, 0.93; 95% CI, 0.89-0.97) (eTable 4 in the Supplement).

Although women who breastfed for less than 6 months had a lower fracture risk than those who never breastfed (any fracture: aHR, 0.97; 95% CI, 0.94-0.99; vertebral fracture: aHR, 0.97; 95% CI, 0.92-1.03), women who breastfed for 12 months or longer had a higher fracture risk (any fracture: aHR, 1.05; 95% CI, 1.03-1.08; vertebral fracture: aHR, 1.22; 95% CI, 1.17-1.27) (eTables 1 and 2 in the Supplement and Figure 3). Hip fracture risk was negatively associated with duration of breastfeeding (<6 months: aHR, 0.84; 95% CI, 0.73-0.97; 6-11 months: aHR, 0.85; 95% CI, 0.76-0.95; and ≥12 months: aHR, 0.84; 95% CI, 0.76-0.93) (eTable 3 in the Supplement and Figure 4).

#### HT and OC Use

Hormone therapy for 5 years or longer was associated with a lower risk of any fracture (aHR, 0.85; 95% CI, 0.83-0.88) (eTable 1 in the Supplement). In model 2, when compared with the never-HT group, HT users had a lower risk of vertebral fracture (HT for <2 years: aHR, 0.95; 95% CI, 0.92-0.98; HT for 2-4 years: aHR, 0.88; 95% CI, 0.84-0.92; and HT for ≥5 years: aHR, 0.82; 95% CI, 0.78-0.86) (eTable 2 in the Supplement and Figure 3). The results were largely consistent across various types of fracture (eTables 1, 3, and 4 in the Supplement and Figure 4). Fracture risk tended to increase with OC use for 1 year or longer (any fracture: aHR, 1.03; 95% CI, 1.01-1.05; vertebral fracture: aHR, 1.06; 95% CI, 1.03-1.09; hip fracture: aHR, 1.06; 95% CI, 0.97-1.15; and other fracture: aHR, 1.03; 95% CI, 1.00-1.02).

### Other Fracture Risk Factors

Current smokers (aHR, 1.06; 95% CI, 1.03-1.09), heavy drinkers (alcohol consumption, ≥30 g/d; aHR, 1.26; 95% CI, 1.19-1.35), and patients with diabetes (aHR, 1.05; 95% CI, 1.03-1.06) were at higher risk of any fracture (eTable 5 in the Supplement). Those who engaged in regular exercise (aHR, 0.98; 95% CI, 0.97-0.99) and were severely obese (defined as a body mass index ≥30 [calculated as weight in kilograms divided by height in meters squared]) (aHR, 0.94; 95% CI, 0.92-0.96) were at lower risk of any fracture.

## Discussion

This study found that female reproductive factors were independently associated with the incidence of fractures among postmenopausal women. Later menarche, earlier menopause, and shorter reproductive span were each independently associated with fracture risk in postmenopausal women. Parous women were found to have a lower risk of any fracture than nulliparous women. Although a breastfeeding duration of 12 months or longer was associated with a higher risk of any fractures and vertebral fractures, breastfeeding was associated with a lower risk of hip fracture. The use of HT was independently associated with a lower risk of fracture in postmenopausal women, but the use of OCs for 1 year or longer was associated with a higher risk of fracture.

Endogenous estrogen exposure occurs mainly during the reproductive phase, which is bounded by menarche and menopause. Lack of estrogen increases bone resorption and decreases the deposition of new bone that normally takes place in weight-bearing bones.^39^ The present study showed significant inverse associations between increased endogenous estrogen exposure and risk of all fracture sites combined, as well as vertebral and hip fractures specifically. Consistent with our findings, one study suggested that earlier menopausal age may be a risk factor associated with decreased whole-body, total-spine, and hip BMD.^10^ In a systematic review of anatomical sites associated with osteoporotic fractures, femoral neck and vertebral fractures were found to be the fracture types most strongly associated with osteoporosis.^36^ The present study provides further evidence that female reproductive factors may be associated with osteoporosis-related fractures.

According to a meta-analysis, increasing parity is associated with a linear reduction in osteoporotic fracture risk among postmenopausal women.^40^ Serum estrogen levels increase during pregnancy to levels approximately 20 to 30 times above their peak during the normal menstrual cycle. Such markedly increased estrogen exposure during each pregnancy may reduce a woman’s fracture risk. Some nulliparous women could be subfertile, producing less estrogen during the menstrual cycle than more fertile women and hence be at greater risk of fracture.^41^ In the present study, primiparous women had a lower risk of any fracture and vertebral fracture than nulliparous women, but multiparity failed to show a significant association with risk of any fracture or vertebral fracture. We also failed to find any significant association between parity and hip fracture. In addition to parity, a shorter interpregnancy interval may have a detrimental association with BMD in postmenopausal age.^42^ In the case of multiparity, it is more likely for age at last childbirth to be older, and postmenopausal women of older age at last childbirth are at increased risk of osteoporosis.^43^ However, this information was not available in the NHIS database.

Although it is believed that bone loss during lactation is restored 6 to 12 months after weaning through mechanisms that remain unclear, we found that prolonged breastfeeding duration increased the risk of any fracture as well as vertebral fracture. In support of our finding, 1 study reported that breastfeeding for only 6 months resulted in a reduction in bone density that stopped after 6 months and returned to previous levels after another 6 months, whereas bone density did not return to original levels when breastfeeding lasted 12 months or longer.^44^ However, a reduced risk of hip fracture in women who breastfed was also noted. Consistent with these findings, a recent meta-analysis reported that the incidence of osteoporotic hip fracture decreased with the extension of breastfeeding time.^14^ Other researchers have reported an association between longer duration of lactation and lower BMD in the lumbar spine but not in the femoral neck or the total hip.^45,46^ Skeletal loss is more profound from trabecular bone than from cortical bone during lactation: the vertebra is primarily trabecular bone, whereas hip bones are largely cortical bone.^47^ Trabecular bone is more metabolically active than cortical bone^48,49^ and possibly more susceptible than cortical bone to hormonal influences and reductions in calcium reserve.^45^ In an animal model, mechanical properties in cortical bone of the femur were substantially improved during the postlactation period compared with those observed at the end of lactation,^50^ and these gains during the postlactation period were also greater than the normal growth observed in control rats, indicating that the postlactation period is an “anabolic” phase for the accumulation of bone mass.^51^ Furthermore, our results suggest that there is something unique about breastfeeding that protects against future fracture at weight-bearing sites because breastfeeding had the greatest association with reducing hip fractures.

We also showed that exogenous hormonal exposure, in particular HT, was associated with a lower risk of fractures. Based on available evidence, the benefits of postmenopausal estrogen therapy for fracture risk are well established.^20,21^ Hormone therapy is associated with a reduced risk of total, hip, and vertebral fractures; however, it may also have adverse effects.^21^ Although the mechanism underlying the association of HT with reduced fracture risk is unclear, it is thought to involve the inhibition of osteoclasts,^52,53^ resulting in decreased bone turnover and improved balance between bone formation and resorption.^52^ Hormone therapy also improves calcium retention through increased intestinal calcium absorption and renal calcium reabsorption.^54^ Our findings support the hypothesis that exogenous female hormone use may protect against future fracture through the beneficial effects of estrogen on bone metabolism.

Oral contraceptives may also improve bone density by biologically plausible mechanisms, but considerable controversy exists as to whether OCs have a positive association with bone density. Furthermore, relatively few studies have investigated the association between prior OC use and fracture in postmenopausal women. In those studies, fracture risk among women who used OCs after 35 to 40 years of age was decreased, suggesting that the association of OC use with later fracture risk depends on a woman’s age at the time of OC use.^55,56^ Our finding of a small but statistically significantly increased risk of fracture among those who had previously used OCs for 1 year or more was unexpected. Similarly, a cohort study of postmenopausal women reported that past OC use for 5 years led to a 15% increased risk of self-reported fracture.^57^ However, because exposure to OCs was determined by participant recall, an apparent decrease in previous use of OCs among older women in our study may have been a result of poor recall. In fact, women with unknown OC use accounted for approximately 5% of the total population, and this segment of the population had a 6% increased risk of vertebral fracture. The exact mechanism underlying this finding needs further investigation.

To our knowledge, this is the largest study performed to date to assess the association between female reproductive factors and fracture, especially according to skeletal site. We directly assessed fracture risk as a clinical outcome rather than BMD, which is a surrogate marker. Furthermore, we examined various reproductive factors comprehensively. The clinical implications of our study are that menstrual and reproductive factors associated with reproductive hormonal disturbances, in particular late menarche, early menopause, and longer reproductive span, are potential risk factors for osteoporosis-related fractures. The World Health Organization fracture-risk (FRAX) tool^58^ is widely used to estimate osteoporotic fractures by integrating clinical risk factors. Currently, the FRAX algorithm does not include reproductive factors, but it is hoped that these factors will be incorporated into existing algorithms as additional supporting data become available in the future.

### Limitations

Our study had several limitations. First, the exposure variables of interest were based on a self-administered questionnaire; therefore, we cannot exclude the possibility of bias caused by inaccurate recall. Second, detailed information about female hormone use, such as age at use or dose, was not available. Further detailed information about female hormone use and users’ potential risk factors for fracture is required to clarify the possible association between exogenous female hormone use and risk of fracture. Third, we were unable to obtain information on the cause of fractures. However, we limited our analysis to common sites of osteoporotic fracture. In addition, our data did not include fractures from traffic accidents, violence, or self-injury, which are not included in NHIS claims data. There is also no reason to believe that traumatic fractures selectively occur by reproductive factors. Fourth, other potential confounders, such as nutritional factors (eg, calcium intake or vitamin D level) or osteoporosis-related factors (eg, osteoporosis treatment or causes of secondary osteoporosis), may have been present. Fifth, there might have been a birth cohort effect owing to improvements in health status and economic growth and rapid assimilation of a Western lifestyle. In Korea, mean age at menarche decreased from 16.9 years for women born between 1920 and 1925 to 13.8 years for those born between 1980 and 1985,^59^ whereas the mean age at menopause increased from 47.9 years for women born in 1929 or earlier to 50.0 years for those born between 1945 and 1949.^60^ As a result, the short reproductive span observed in this study may primarily reflect the characteristics of the older participants in the cohort. The association of age with reproductive factors, however, was minimized by controlling for age. Sixth, this was a retrospective study, and the findings should be interpreted as associations, not as causality.

## Conclusions

The findings of this large, population-based cohort study suggest that female reproductive factors are independently associated with fracture development. An association was also noted between lower lifetime endogenous estrogen exposure and increased fracture incidence.

## Notes

Supplement. **eMethods.** Supplemental Methods **eTable 1.** Hazard Ratios and 95% Confidence Intervals of Any Fracture According to Reproductive Factors **eTable 2.** Hazard Ratios and 95% Confidence Intervals of Vertebral Fracture According to Reproductive Factors **eTable 3.** Hazard Ratios and 95% Confidence Intervals of Hip Fracture According to Reproductive Factors **eTable 4.** Hazard Ratios and 95% Confidence Intervals of Other Fractures According to Reproductive Factors **eTable 5.** Hazard Ratios and 95% Confidence Intervals of Fractures **eFigure.** Flow Chart of the Study Population

## References

[1] Kanis JA Diagnosis of osteoporosis and assessment of fracture risk. Lancet. 2002;359(9321):1929-1936. doi:10.1016/S0140-6736(02)08761-5 12057569

[2] Kang HY, Yang KH, Kim YN, Incidence and mortality of hip fracture among the elderly population in South Korea: a population-based study using the National Health Insurance claims data. BMC Public Health. 2010;10:230. doi:10.1186/1471-2458-10-230 20438644 PMC2874780

[3] Zhang Q, Greenbaum J, Zhang WD, Sun CQ, Deng HW Age at menarche and osteoporosis: a mendelian randomization study. Bone. 2018;117:91-97. doi:10.1016/j.bone.2018.09.015 30240960 PMC6346741

[4] Johnell O, Gullberg B, Kanis JA, Risk factors for hip fracture in European women: the MEDOS Study. Mediterranean Osteoporosis Study. J Bone Miner Res. 1995;10(11):1802-1815. doi:10.1002/jbmr.5650101125 8592959

[5] Shimizu Y, Sawada N, Nakamura K, ; JPHC Study Group Menstrual and reproductive factors and risk of vertebral fractures in Japanese women: the Japan Public Health Center-based prospective (JPHC) study. Osteoporos Int. 2018;29(12):2791-2801. doi:10.1007/s00198-018-4665-8 30143851

[6] van Der Voort DJ, van Der Weijer PH, Barentsen R Early menopause: increased fracture risk at older age. Osteoporos Int. 2003;14(6):525-530. doi:10.1007/s00198-003-1408-1 12730751

[7] Lespessailles E, Cotté FE, Roux C, Fardellone P, Mercier F, Gaudin AF Prevalence and features of osteoporosis in the French general population: the Instant Study. Joint Bone Spine. 2009;76(4):394-400. doi:10.1016/j.jbspin.2008.10.008 19297229

[8] Peng K, Yao P, Kartsonaki C, ; China Kadoorie Biobank Collaborative Group Menopause and risk of hip fracture in middle-aged Chinese women: a 10-year follow-up of China Kadoorie Biobank. Menopause. 2020;27(3):311-318. doi:10.1097/GME.0000000000001478 31876618 PMC7616980

[9] Svejme O, Ahlborg HG, Nilsson JÅ, Karlsson MK Early menopause and risk of osteoporosis, fracture and mortality: a 34-year prospective observational study in 390 women. BJOG. 2012;119(7):810-816. doi:10.1111/j.1471-0528.2012.03324.x 22531019

[10] Sullivan SD, Lehman A, Thomas F, Effects of self-reported age at nonsurgical menopause on time to first fracture and bone mineral density in the Women’s Health Initiative Observational Study. Menopause. 2015;22(10):1035-1044. doi:10.1097/GME.0000000000000451 25803670 PMC4580482

[11] Bonjour JP, Chevalley T Pubertal timing, bone acquisition, and risk of fracture throughout life. Endocr Rev. 2014;35(5):820-847. doi:10.1210/er.2014-1007 25153348

[12] Nam HS, Kweon SS, Choi JS, Racial/ethnic differences in bone mineral density among older women. J Bone Miner Metab. 2013;31(2):190-198. doi:10.1007/s00774-012-0402-0 23143509 PMC4109723

[13] Duan X, Wang J, Jiang X A meta-analysis of breastfeeding and osteoporotic fracture risk in the females. Osteoporos Int. 2017;28(2):495-503. doi:10.1007/s00198-016-3753-x 27577724

[14] Xiao H, Zhou Q, Niu G, Association between breastfeeding and osteoporotic hip fracture in women: a dose-response meta-analysis. J Orthop Surg Res. 2020;15(1):15. doi:10.1186/s13018-019-1541-y 31948457 PMC6966889

[15] Bjørnerem A, Ahmed LA, Jørgensen L, Størmer J, Joakimsen RM Breastfeeding protects against hip fracture in postmenopausal women: the Tromsø study. J Bone Miner Res. 2011;26(12):2843-2850. doi:10.1002/jbmr.496 21898594

[16] Bolzetta F, Veronese N, De Rui M, Duration of breastfeeding as a risk factor for vertebral fractures. Bone. 2014;68:41-45. doi:10.1016/j.bone.2014.08.001 25120256

[17] Ahn SK, Kam S, Chun BY Incidence of and factors for self-reported fragility fractures among middle-aged and elderly women in rural Korea: an 11-year follow-up study. J Prev Med Public Health. 2014;47(6):289-297. doi:10.3961/14.020 25274003 PMC4263002

[18] Cauley JA, Wu L, Wampler NS, Clinical risk factors for fractures in multi-ethnic women: the Women’s Health Initiative. J Bone Miner Res. 2007;22(11):1816-1826. doi:10.1359/jbmr.070713 17638574

[19] Torgerson DJ, Bell-Syer SE Hormone replacement therapy and prevention of nonvertebral fractures: a meta-analysis of randomized trials. JAMA. 2001;285(22):2891-2897. doi:10.1001/jama.285.22.2891 11401611

[20] Rossouw JE, Anderson GL, Prentice RL, ; Writing Group for the Women’s Health Initiative Investigators Risks and benefits of estrogen plus progestin in healthy postmenopausal women: principal results from the Women’s Health Initiative randomized controlled trial. JAMA. 2002;288(3):321-333. doi:10.1001/jama.288.3.321 12117397

[21] Zhu L, Jiang X, Sun Y, Shu W Effect of hormone therapy on the risk of bone fractures: a systematic review and meta-analysis of randomized controlled trials. Menopause. 2016;23(4):461-470. doi:10.1097/GME.0000000000000519 26529613

[22] Lopez LM, Grimes DA, Schulz KF, Curtis KM, Chen M Steroidal contraceptives: effect on bone fractures in women. Cochrane Database Syst Rev. 2014;(6):CD006033. doi:10.1002/14651858.CD006033.pub5 24960023 PMC11127753

[23] Gambacciani M, Monteleone P, Ciaponi M, Sacco A, Genazzani AR Effects of oral contraceptives on bone mineral density. Treat Endocrinol. 2004;3(3):191-196. doi:10.2165/00024677-200403030-00006 16026114

[24] Pasco JA, Kotowicz MA, Henry MJ, Panahi S, Seeman E, Nicholson GC Oral contraceptives and bone mineral density: a population-based study. Am J Obstet Gynecol. 2000;182(2):265-269. doi:10.1016/S0002-9378(00)70209-2 10694322

[25] Wei S, Jones G, Thomson R, Dwyer T, Venn A Oral contraceptive use and bone mass in women aged 26-36 years. Osteoporos Int. 2011;22(1):351-355. doi:10.1007/s00198-010-1180-y 20195845

[26] Reed SD, Scholes D, LaCroix AZ, Ichikawa LE, Barlow WE, Ott SM Longitudinal changes in bone density in relation to oral contraceptive use. Contraception. 2003;68(3):177-182. doi:10.1016/S0010-7824(03)00147-1 14561537

[27] Wanichsetakul P, Kamudhamas A, Watanaruangkovit P, Siripakarn Y, Visutakul P Bone mineral density at various anatomic bone sites in women receiving combined oral contraceptives and depot-medroxyprogesterone acetate for contraception. Contraception. 2002;65(6):407-410. doi:10.1016/S0010-7824(02)00308-6 12127638

[28] Allali F, El Mansouri L, Abourazzak Fz, The effect of past use of oral contraceptive on bone mineral density, bone biochemical markers and muscle strength in healthy pre and post menopausal women. BMC Womens Health. 2009;9:31. doi:10.1186/1472-6874-9-31 19887010 PMC2776575

[29] Prior JC, Kirkland SA, Joseph L, Oral contraceptive use and bone mineral density in premenopausal women: cross-sectional, population-based data from the Canadian Multicentre Osteoporosis Study. CMAJ. 2001;165(8):1023-1029. 11699697 PMC81536

[30] Scholes D, LaCroix AZ, Hubbard RA, Oral contraceptive use and fracture risk around the menopausal transition. Menopause. 2016;23(2):166-174. doi:10.1097/GME.0000000000000595 26757274 PMC4731309

[31] Wei S, Venn A, Ding C, Foley S, Laslett L, Jones G The association between oral contraceptive use, bone mineral density and fractures in women aged 50-80 years. Contraception. 2011;84(4):357-362. doi:10.1016/j.contraception.2011.02.001 21920189

[32] Lee J, Lee JS, Park SH, Shin SA, Kim K Cohort profile: the National Health Insurance Service–National Sample Cohort (NHIS-NSC), South Korea. Int J Epidemiol. 2017;46(2):e15. doi:10.1093/ije/dyv319 26822938

[33] Lee YH, Han K, Ko SH, Ko KS, Lee KU; Taskforce Team of Diabetes Fact Sheet of the Korean Diabetes Association Data analytic process of a nationwide population-based study using national health information database established by National Health Insurance Service. Diabetes Metab J. 2016;40(1):79-82. doi:10.4093/dmj.2016.40.1.79 26912157 PMC4768054

[34] Equator Network. The Strengthening the Reporting of Observational Studies in Epidemiology (STROBE) statement: guidelines for reporting observational studies. Updated October 22, 2019. Accessed October 13, 2020. https://www.equator-network.org/reporting-guidelines/strobe/

[35] Yoo KY Cancer control activities in the Republic of Korea. Jpn J Clin Oncol. 2008;38(5):327-333. doi:10.1093/jjco/hyn026 18407932

[36] Warriner AH, Patkar NM, Curtis JR, Which fractures are most attributable to osteoporosis? J Clin Epidemiol. 2011;64(1):46-53. doi:10.1016/j.jclinepi.2010.07.007 21130353 PMC5030717

[37] Lix LM, Azimaee M, Osman BA, Osteoporosis-related fracture case definitions for population-based administrative data. BMC Public Health. 2012;12:301. doi:10.1186/1471-2458-12-301 22537071 PMC3356235

[38] Kim HY, Ha YC, Kim TY, Healthcare costs of osteoporotic fracture in Korea: information from the National Health Insurance Claims Database, 2008-2011. J Bone Metab. 2017;24(2):125-133. doi:10.11005/jbm.2017.24.2.125 28642857 PMC5472799

[39] Raisz LG Pathogenesis of osteoporosis: concepts, conflicts, and prospects. J Clin Invest. 2005;115(12):3318-3325. doi:10.1172/JCI27071 16322775 PMC1297264

[40] Wang Q, Huang Q, Zeng Y, Parity and osteoporotic fracture risk in postmenopausal women: a dose-response meta-analysis of prospective studies. Osteoporos Int. 2016;27(1):319-330. doi:10.1007/s00198-015-3351-3 26439242

[41] Hillier TA, Rizzo JH, Pedula KL, Nulliparity and fracture risk in older women: the study of osteoporotic fractures. J Bone Miner Res. 2003;18(5):893-899. doi:10.1359/jbmr.2003.18.5.893 12733729

[42] Sahin Ersoy G, Giray B, Subas S, Interpregnancy interval as a risk factor for postmenopausal osteoporosis. Maturitas. 2015;82(2):236-240. doi:10.1016/j.maturitas.2015.07.014 26254682

[43] We JS, Han K, Kwon HS, Kil K Effect of childbirth age on bone mineral density in postmenopausal women. J Korean Med Sci. 2018;33(48):e311. doi:10.3346/jkms.2018.33.e311 30473652 PMC6249168

[44] More C, Bettembuk P, Bhattoa HP, Balogh A The effects of pregnancy and lactation on bone mineral density. Osteoporos Int. 2001;12(9):732-737. doi:10.1007/s001980170048 11605738

[45] Mori T, Ishii S, Greendale GA, Parity, lactation, bone strength, and 16-year fracture risk in adult women: findings from the Study of Women’s Health Across the Nation (SWAN). Bone. 2015;73:160-166. doi:10.1016/j.bone.2014.12.013 25528102 PMC4364696

[46] Tsvetov G, Levy S, Benbassat C, Shraga-Slutzky I, Hirsch D Influence of number of deliveries and total breast-feeding time on bone mineral density in premenopausal and young postmenopausal women. Maturitas. 2014;77(3):249-254. doi:10.1016/j.maturitas.2013.11.003 24332872

[47] Kirby BJ, Ardeshirpour L, Woodrow JP, Skeletal recovery after weaning does not require PTHrP. J Bone Miner Res. 2011;26(6):1242-1251. doi:10.1002/jbmr.339 21308774 PMC3179289

[48] Miyamoto T, Miyakoshi K, Sato Y, Changes in bone metabolic profile associated with pregnancy or lactation. Sci Rep. 2019;9(1):6787. doi:10.1038/s41598-019-43049-1 31086225 PMC6513862

[49] Greendale GA, Sowers M, Han W, Bone mineral density loss in relation to the final menstrual period in a multiethnic cohort: results from the Study of Women’s Health Across the Nation (SWAN). J Bone Miner Res. 2012;27(1):111-118. doi:10.1002/jbmr.534 21976317 PMC3378821

[50] Vajda EG, Bowman BM, Miller SC Cancellous and cortical bone mechanical properties and tissue dynamics during pregnancy, lactation, and postlactation in the rat. Biol Reprod. 2001;65(3):689-695. doi:10.1095/biolreprod65.3.689 11514329

[51] Bowman BM, Miller SC Skeletal mass, chemistry, and growth during and after multiple reproductive cycles in the rat. Bone. 1999;25(5):553-559. doi:10.1016/S8756-3282(99)00204-5 10574575

[52] Hillard TC, Stevenson JC Role of oestrogen in the development of osteoporosis. Calcif Tissue Int. 1991;49(suppl):S55-S59. doi:10.1007/BF02555090 1933600

[53] Rizzoli R, Bonjour JP Hormones and bones. Lancet. 1997;349(suppl 1):sI20-sI23. doi:10.1016/S0140-6736(97)90007-6 9057775

[54] Michaëlsson K, Baron JA, Farahmand BY, ; Swedish Hip Fracture Study Group Hormone replacement therapy and risk of hip fracture: population based case-control study. BMJ. 1998;316(7148):1858-1863. doi:10.1136/bmj.316.7148.1858 9632404 PMC28583

[55] Michaëlsson K, Baron JA, Farahmand BY, Ljunghall S Use of low potency estrogens does not reduce the risk of hip fracture. Bone. 2002;30(4):613-618. doi:10.1016/S8756-3282(01)00701-3 11934654

[56] Cooper C, Hannaford P, Croft P, Kay CR Oral contraceptive pill use and fractures in women: a prospective study. Bone. 1993;14(1):41-45. doi:10.1016/8756-3282(93)90254-8 8443001

[57] Barad D, Kooperberg C, Wactawski-Wende J, Liu J, Hendrix SL, Watts NB Prior oral contraception and postmenopausal fracture: a Women’s Health Initiative observational cohort study. Fertil Steril. 2005;84(2):374-383. doi:10.1016/j.fertnstert.2005.01.132 16084878

[58] Kanis JA, Johnell O, Oden A, Johansson H, McCloskey E FRAX and the assessment of fracture probability in men and women from the UK. Osteoporos Int. 2008;19(4):385-397. doi:10.1007/s00198-007-0543-5 18292978 PMC2267485

[59] Cho GJ, Park HT, Shin JH, Age at menarche in a Korean population: secular trends and influencing factors. Eur J Pediatr. 2010;169(1):89-94. doi:10.1007/s00431-009-0993-1 19504269

[60] Park CY, Lim JY, Park HY Age at natural menopause in Koreans: secular trends and influences thereon. Menopause. 2018;25(4):423-429. doi:10.1097/GME.0000000000001019 29112598

