# Supplementary material for: Association of Female Reproductive Factors With Incidence of Fracture Among Postmenopausal Women in Korea
Source: JAMA Netw Open. 2021 Jan 6;4(1):e2030405. doi: 10.1001/jamanetworkopen.2020.30405 (PMC7788464; doi:10.1001/jamanetworkopen.2020.30405)
Supplement: Supplement. — eMethods. Supplemental Methods eTable 1. Hazard Ratios and 95% Confidence Intervals of Any Fracture According to Reproductive Factors eTable 2. Hazard Ratios and 95% Confidence Intervals of Vertebral Fracture According to Reproductive Factors eTable 3. Hazard Ratios and 95% Confidence Intervals of Hip Fracture According to Reproductive Factors eTable 4. Hazard Ratios and 95% Confidence Intervals of Other Fractures According to Reproductive Factors eTable 5. Hazard Ratios and 95% Confidence Intervals of Fractures eFigure. Flow Chart of the Study Population [file jamanetwopen-e2030405-s001.pdf]

Supplementary Online Content

Yoo JE, Shin DW, Han K, Kim D, Yoon JW, Lee DY. Association of female reproductive factors with incidence of fracture among postmenopausal women in Korea. *JAMA Netw Open.* 2021;4(1):e2030405. doi:10.1001/jamanetworkopen.2020.30405

eMethods. Supplemental Methods

eTable 1. Hazard Ratios and 95% Confidence Intervals of Any Fracture According to Reproductive Factors

eTable 2. Hazard Ratios and 95% Confidence Intervals of Vertebral Fracture According to Reproductive Factors

eTable 3. Hazard Ratios and 95% Confidence Intervals of Hip Fracture According to Reproductive Factors

eTable 4. Hazard Ratios and 95% Confidence Intervals of Other Fractures According to Reproductive Factors

eTable 5. Hazard Ratios and 95% Confidence Intervals of Fractures

eFigure. Flow Chart of the Study Population

This supplementary material has been provided by the authors to give readers additional information about their work.

© 2021 Yoo JE et al. *JAMA Network Open.*

eMethods. Supplemental Methods

Exposure: Reproductive factors

During National Cancer Screening Program, information about age at menarche, age at menopause, parity, total lifetime breast feeding history, and use of hormone therapy (HT) / oral contraceptives (OCs) was collected by self-administered questionnaire. Age at menarche was categorized as ≤12 years, 13-14 years, 15-16 years, and ≥17 years, to be consistent with the distribution of age at menarche among Korean women. Age at menopause was categorized as <40 years, 40-44 years, 45-49 years, 50-54 years, and ≥55 years. Reproductive span was calculated as the interval between the age at menarche and the age at menopause. Parity was categorized as 0 (nulliparous), 1 (primiparous), or ≥2 (multiparous). Total lifetime breast-feeding history was categorized as never, <6 months, 6-11 months or ≥12 total months. The duration of HT was categorized as never, <2 years, 2-4 years, ≥5 years, or unknown. The duration of OC use was categorized as never, <1 years, ≥1 years, or unknown.

Study outcome: Fractures

The primary endpoint was newly diagnosed fractures during the follow-up period. Fractures were defined by ICD-10 codes as follows: (1) vertebral (S22.0, S22.1, S32.0, M48.4 and M48.5), (2) hip (S72.0 and S72.1), or (3) other fractures. Other fractures included clavicle (S42.0), upper arm (S42.2 and S42.3), wrist (S52.5 and S52.6), and ankle (S82.3, S82.5 and S82.6). Vertebral or other fractures were identified when subjects had two or more outpatient visits with relevant diagnosis codes within 12 months. Hip fracture was defined as one hospitalization with the relevant diagnosis code. Any fracture was defined as at least one of these three fracture types.

Covariates

Income level was based on the monthly insurance premium because insurance contribution is determined based on income level and not on health risk in Korea. Subjects were classified as never, ex-, or current smokers. Based on daily alcohol consumption, alcohol consumption was classified as none (0 g/day), moderate (<30 g/day), or heavy (≥30 g/day). Regular exercise was defined as performing >30 minutes of moderate physical activity at least five times per week or >20 minutes of strenuous physical activity at least three times per week. Subjects were categorized into five groups based on body mass index (BMI, kg/m^2) according to the Asia-Pacific criteria of the World Health Organization as follows: underweight (<18.5 kg/m^2), normal (18.5 to <23 kg/m^2), overweight (23 to <25 kg/m^2), obese (25 to <30 kg/m^2), and severely obese (≥30 kg/m^2). Systolic and diastolic blood pressure (BP) were measured in a seated position after at least 5 minutes rest. Blood samples for measurement of serum fasting glucose and lipid levels were drawn after an overnight fast. Baseline comorbidities (hypertension, diabetes mellitus, dyslipidemia, and cancer) were identified based on the combination of past medical history and ICD-10 and prescription codes.

© 2021 Yoo JE et al. *JAMA Network Open.*

eTable 1. Hazard Ratios and 95% Confidence Intervals of Any Fracture According to Reproductive Factors

Reproductive factors	Subjects (N)	Events (n)	Follow-up duration (PYs)	Incidence rate (per 1,000 PYs)	Model 1	Model 2	Model 3
Age at menarche, y							
≤12	12,580	1,285	99,083.4	13.0	1 (ref.)	1 (ref.)	
13-14	159,722	19,250	1,245,522.7	15.5	**1.19 (1.12, 1.26)**	**1.10 (1.04, 1.16)**	
15-16	500,445	70,016	3,853,210.6	18.2	**1.40 (1.32, 1.48)**	**1.16 (1.10, 1.23)**	
≥17	599,368	99,332	4,538,316.8	21.9	**1.69 (1.60, 1.78)**	**1.24 (1.17, 1.31)**	
Age at menopause, y							
<40	21,101	3,748	157,201.0	23.8	1 (ref.)	1 (ref.)	
40-44	71,142	11,861	535,222.2	22.2	**0.93 (0.90, 0.96)**	**0.97 (0.93, 1.01)**	
45-49	345,678	51,249	2,648,260.5	19.4	**0.81 (0.78, 0.84)**	**0.94 (0.91, 0.97)**	
50-54	699,018	101,617	5,364,751.4	18.9	**0.79 (0.77, 0.82)**	**0.90 (0.88, 0.93)**	
≥55	135,176	21,408	1,030,698.4	20.8	**0.87 (0.84, 0.90)**	**0.89 (0.86, 0.93)**	
Reproductive span, y							
<30	170,474	29,718	1,277,414.9	23.3	1 (ref.)		1 (ref.)
30-34	529,589	80,796	4,043,760.9	20.0	**0.86 (0.85, 0.87)**		**0.94 (0.93, 0.95)**
35-39	490,384	67,381	3,788,551.8	17.8	**0.76 (0.75, 0.77)**		**0.89 (0.88, 0.90)**
≥40	81,668	11,988	626,405.9	19.1	**0.82 (0.81, 0.84)**		**0.86 (0.84, 0.88)**
Parity							
Nulliparity	32,006	4,242	247,443.9	17.1	1 (ref.)	1 (ref.)	1 (ref.)
1	79,411	9,446	619,475.1	15.2	**0.89 (0.86, 0.92)**	**0.96 (0.92, 0.99)**	**0.95 (0.92, 0.99)**
≥2	1,160,698	176,195	8,869,214.4	19.9	**1.16 (1.12, 1.20)**	0.99 (0.95, 1.02)	0.98 (0.95, 1.02)
Duration of breast feeding, mo							
Never	86,477	10,449	673,737.5	15.5	1 (ref.)	1 (ref.)	1 (ref.)
<6	86,290	9,844	675,687.5	14.6	**0.94 (0.91, 0.97)**	**0.97 (0.94, 0.99)**	**0.97 (0.94, 0.99)**
6-11	225,607	29,142	1,750,226.8	16.7	**1.07 (1.05, 1.10)**	1.00 (0.97, 1.02)	1.00 (0.98, 1.02)
≥12	873,741	140,448	6,636,481.7	21.2	**1.37 (1.34, 1.39)**	**1.05 (1.03, 1.08)**	**1.06 (1.04, 1.09)**

© 2021 Yoo JE et al. *JAMA Network Open.*

Duration of HT, y							
Never	1,023,519	156,924	7,801,630.3	20.1	1 (ref.)	1 (ref.)	1 (ref.)
<2	118,146	15,351	922,759.7	16.6	**0.83 (0.81, 0.84)**	0.99 (0.97, 1.01)	0.99 (0.97, 1.00)
2-4	48,812	6,113	382,048.7	16.0	**0.80 (0.78, 0.82)**	**0.93 (0.91, 0.95)**	**0.93 (0.91, 0.95)**
≥5	37,581	4,723	294,392.8	16.0	**0.80 (0.77, 0.82)**	**0.85 (0.83, 0.88)**	**0.85 (0.83, 0.88)**
Unknown	44,057	6,772	335,301.9	20.2	1.00 (0.98, 1.03)	1.01 (0.98, 1.04)	1.01 (0.98, 1.04)
Duration of OC use, y							
Never	1,015,265	151,858	7,762,969.8	19.6	1 (ref.)	1 (ref.)	1 (ref.)
<1	116,384	16,603	898,277.1	18.5	**0.95 (0.93,0.96)**	1.00 (0.98, 1.02)	1.00 (0.99, 1.02)
≥1	77,581	11,863	594,891.8	19.9	1.02 (1.00, 1.04)	**1.02 (1.00, 1.04)**	**1.03 (1.01, 1.05)**
Unknown	62,885	9,559	479,994.8	19.9	1.02 (1.00, 1.04)	1.02 (1.00, 1.05)	1.02 (1.00, 1.04)

Abbreviations: PYs, person-years; HT, hormone therapy; OC, oral contraceptive
Model 1: crude model
Model 2: the full model included age, age at menarche, age at menopause, parity, duration of breast feeding, duration of HT, duration of OC use, alcohol consumption, smoking, regular exercise, income, body mass index, hypertension, diabetes, dyslipidemia, and cancer.
Model 3: the full model included reproductive span instead of age at menarche and menopause in Model 2.

© 2021 Yoo JE et al. *JAMA Network Open.*

eTable 2. Hazard Ratios and 95% Confidence Intervals of Vertebral Fracture According to Reproductive Factors

Reproductive factors	Subjects (N)	Events (n)	Follow-up duration (PYs)	Incidence rate (per 1,000 PYs)	Model 1	Model 2	Model 3
Age at menarche, y							
≤12	12,580	357	99,083.4	3.6	1 (ref.)	1 (ref.)	
13-14	159,722	5,899	1,245,522.7	4.7	**1.32 (1.18, 1.46)**	**1.12 (1.01, 1.25)**	
15-16	500,445	25,166	3,853,210.6	6.5	**1.82 (1.64, 2.02)**	**1.26 (1.14, 1.40)**	
≥17	599,368	41,310	4,538,316.8	9.1	**2.53 (2.28, 2.81)**	**1.42 (1.28, 1.58)**	
Age at menopause, y							
<40	21,101	1,764	157,201.0	11.2	1 (ref.)	1 (ref.)	
40-44	71,142	5,285	535,222.2	9.9	**0.88 (0.83, 0.93)**	**0.94 (0.89, 0.99)**	
45-49	345,678	20,155	2,648,260.5	7.6	**0.68 (0.65, 0.71)**	**0.88 (0.84, 0.93)**	
50-54	699,018	37,561	5,364,751.4	7.0	**0.62 (0.59, 0.65)**	**0.81 (0.77, 0.85)**	
≥55	135,176	7,967	1,030,698.4	7.7	**0.69 (0.65, 0.72)**	**0.77 (0.73, 0.81)**	
Reproductive span, y							
<30	170,474	13,411	1,277,414.9	10.5	1 (ref.)		1 (ref.)
30-34	529,589	31,978	4,043,760.9	7.9	**0.75 (0.74, 0.77)**		**0.89 (0.87, 0.91)**
35-39	490,384	23,135	3,788,551.8	6.1	**0.58 (0.57, 0.59)**		**0.79 (0.77, 0.81)**
≥40	81,668	4,208	626,405.9	6.7	**0.64 (0.62, 0.66)**		**0.73 (0.71, 0.76)**
Parity							
Nulliparity	32,006	1,244	247,443.9	5.0	1 (ref.)	1 (ref.)	1 (ref.)
1	79,411	2,579	619,475.1	4.2	**0.83 (0.77, 0.89)**	**0.90 (0.84, 0.97)**	**0.90 (0.84, 0.97)**
≥2	1,160,698	68,909	8,869,214.4	7.8	**1.55 (1.46, 1.64)**	**1.07 (1.00, 1.14)**	1.06 (1.00, 1.13)
Duration of breast feeding, mo							
Never	86,477	2,889	673,737.5	4.3	1 (ref.)	1 (ref.)	1 (ref.)
<6	86,290	2,747	675,687.5	4.1	**0.95 (0.90, 1.00)**	0.97 (0.92, 1.03)	0.97 (0.92, 1.02)
6-11	225,607	9,303	1,750,226.8	5.3	**1.24 (1.19, 1.29)**	1.03 (0.99, 1.08)	1.04 (0.99, 1.09)
≥12	873,741	57,793	6,636,481.7	8.7	**2.03 (1.96, 2.11)**	**1.22 (1.17, 1.27)**	**1.23 (1.18, 1.29)**

© 2021 Yoo JE et al. *JAMA Network Open.*

Duration of HT, y							
Never	1,023,519	61,863	7,801,630.3	7.9	1 (ref.)	1 (ref.)	1 (ref.)
<2	118,146	4,829	922,759.7	5.2	**0.66 (0.64, 0.68)**	**0.95 (0.92, 0.98)**	**0.95 (0.92, 0.98)**
2-4	48,812	1,893	382,048.7	5.0	**0.62 (0.60, 0.65)**	**0.88 (0.84, 0.92)**	**0.88 (0.84, 0.92)**
≥5	37,581	1,570	294,392.8	5.3	**0.67 (0.64, 0.71)**	**0.82 (0.78, 0.86)**	**0.81 (0.77, 0.86)**
Unknown	44,057	2,577	335,301.9	7.7	0.97 (0.93, 1.01)	0.98 (0.94, 1.03)	0.98 (0.94, 1.03)
Duration of OC use, y							
Never	1,015,265	58,466	7,762,969.8	7.5	1 (ref.)	1 (ref.)	1 (ref.)
<1	116,384	6,039	898,277.1	6.7	**0.89 (0.87, 0.92)**	1.01 (0.99, 1.04)	1.02 (0.99, 1.04)
≥1	77,581	4,552	594,891.8	7.7	1.02 (0.99, 1.05)	**1.06 (1.03, 1.09)**	**1.06 (1.03, 1.10)**
Unknown	62,885	3,675	479,994.8	7.7	1.02 (0.98, 1.05)	**1.06 (1.02, 1.10)**	**1.05 (1.01, 1.09)**

Abbreviations: PYs, person-years; HT, hormone therapy; OC, oral contraceptive
Model 1: crude model
Model 2: the full model included age, age at menarche, age at menopause, parity, duration of breast feeding, duration of HT, duration of OC use, alcohol consumption, smoking, regular exercise, income, body mass index, hypertension, diabetes, dyslipidemia, and cancer.
Model 3: the full model included reproductive span instead of age at menarche and menopause in Model 2.

© 2021 Yoo JE et al. *JAMA Network Open.*

eTable 3. Hazard Ratios and 95% Confidence Intervals of Hip Fracture According to Reproductive Factors

Reproductive factors	Subjects (N)	Events (n)	Follow-up duration (PYs)	Incidence rate (per 1,000 PYs)	Model 1	Model 2	Model 3
Age at menarche, y							
≤12	12,580	47	99,083.4	0.5	1 (ref.)	1 (ref.)	
13-14	159,722	886	1,245,522.7	0.7	**1.50 (1.12, 2.01)**	1.18 (0.88, 1.58)	
15-16	500,445	3,845	3,853,210.6	1.0	**2.10 (1.58, 2.80)**	1.18 (0.89, 1.58)	
≥17	599,368	6,375	4,538,316.8	1.4	**2.96 (2.22, 3.94)**	1.23 (0.93, 1.64)	
Age at menopause, y							
<40	21,101	300	157,201.0	1.9	1 (ref.)	1 (ref.)	
40-44	71,142	905	535,222.2	1.7	**0.89 (0.78, 1.01)**	0.95 (0.84, 1.09)	
45-49	345,678	3,052	2,648,260.5	1.2	**0.60 (0.54, 0.68)**	0.91 (0.81, 1.03)	
50-54	699,018	5,622	5,364,751.4	1.0	**0.55(0.49, 0.62)**	**0.86 (0.77, 0.97)**	
≥55	135,176	1,274	1,030,698.4	1.2	**0.65 (0.57, 0.74)**	0.88 (0.78, 1.00)	
Reproductive span, y							
<30	170,474	2,206	1,277,414.9	1.7	1 (ref.)		1 (ref.)
30-34	529,589	4,964	4,043,760.9	1.2	**0.71 (0.68, 0.75)**		**0.93 (0.88, 0.98)**
35-39	490,384	3,301	3,788,551.8	0.9	**0.51 (0.48, 0.53)**		**0.88 (0.83, 0.93)**
≥40	81,668	682	626,405.9	1.1	**0.63 (0.58, 0.69)**		**0.87 (0.80, 0.95)**
Parity							
Nulliparity	32,006	202	247,443.9	0.8	1 (ref.)	1 (ref.)	1 (ref.)
1	79,411	439	619,475.1	0.7	0.87 (0.73, 1.02)	1.13 (0.95, 1.34)	1.12 (0.95, 1.34)
≥2	1,160,698	10,512	8,869,214.4	1.2	**1.45 (1.26, 1.66)**	1.01 (0.87, 1.19)	1.01 (0.87, 1.18)
Duration of breast feeding, mo							
Never	86,477	494	673,737.5	0.7	1 (ref.)	1 (ref.)	1 (ref.)
<6	86,290	385	675,687.5	0.6	**0.78 (0.68, 0.89)**	**0.84 (0.73, 0.97)**	**0.84 (0.73, 0.97)**
6-11	225,607	1,393	1,750,226.8	0.8	1.08 (0.98, 1.20)	**0.85 (0.76, 0.95)**	**0.85 (0.76, 0.95)**
≥12	873,741	8,881	6,636,481.7	1.3	**1.82 (1.66, 1.99)**	**0.84 (0.76, 0.93)**	**0.84 (0.76, 0.93)**

© 2021 Yoo JE et al. *JAMA Network Open.*

Duration of HT, y							
Never	1,023,519	9,816	7,801,630.3	1.3	1 (ref.)	1 (ref.)	1 (ref.)
<2	118,146	522	922,759.7	0.6	**0.45 (0.41, 0.49)**	**0.90 (0.82, 0.98)**	**0.90 (0.82, 0.98)**
2-4	48,812	198	382,048.7	0.5	**0.41 (0.36, 0.47)**	**0.80 (0.69, 0.92)**	**0.80 (0.69, 0.92)**
≥5	37,581	202	294,392.8	0.7	**0.54 (0.47, 0.63)**	**0.86 (0.74, 0.99)**	**0.86 (0.75, 0.99)**
Unknown	44,057	415	335,301.9	1.2	0.99 (0.89, 1.09)	0.98 (0.88, 1.09)	0.98 (0.88, 1.09)
Duration of OC use, y							
Never	1,015,265	9,159	7,762,969.8	1.2	1 (ref.)	1 (ref.)	1 (ref.)
<1	116,384	776	898,277.1	0.9	**0.73 (0.68, 0.79)**	0.98 (0.91, 1.06)	0.99 (0.92, 1.06)
≥1	77,581	637	594,891.8	1.1	**0.91 (0.84, 0.99)**	1.06 (0.97, 1.15)	1.06 (0.98, 1.15)
Unknown	62,885	581	479,994.8	1.2	1.03 (0.95, 1.12)	1.03 (0.94, 1.13)	1.03 (0.94, 1.13)

Abbreviations: PYs, person-years; HT, hormone therapy; OC, oral contraceptive
Model 1: crude model
Model 2: the full model included age, age at menarche, age at menopause, parity, duration of breast feeding, duration of HT, duration of OC use, alcohol consumption, smoking, regular exercise, income, body mass index, hypertension, diabetes, dyslipidemia, and cancer.
Model 3: the full model included reproductive span instead of age at menarche and menopause in Model 2.

© 2021 Yoo JE et al. *JAMA Network Open.*

eTable 4. Hazard Ratios and 95% Confidence Intervals of Other Fractures According to Reproductive Factors

Reproductive factors	Subjects (N)	Events (n)	Follow-up duration (PYs)	Incidence rate (per 1,000 PYs)	Model 1	Model 2	Model 3
Age at menarche, y							
≤12	12,580	887	99,083.4	9.0	1 (ref.)	1 (ref.)	
13-14	159,722	12,530	1,245,522.7	10.1	**1.12 (1.05, 1.20)**	**1.10 (1.03, 1.18)**	
15-16	500,445	41,357	3,853,210.6	10.7	**1.20 (1.12, 1.28)**	**1.15 (1.07, 1.23)**	
≥17	599,368	52,121	4,538,316.8	11.5	**1.28 (1.20, 1.37)**	**1.19 (1.12, 1.28)**	
Age at menopause, y							
<40	21,101	1,707	157,201.0	10.9	1 (ref.)	1 (ref.)	
40-44	71,142	5,728	535,222.2	10.7	0.99 (0.93, 1.04)	1.00 (0.94, 1.05)	
45-49	345,678	28,286	2,648,260.5	10.7	0.98 (0.94, 1.03)	1.01 (0.97, 1.07)	
50-54	699,018	58,917	5,364,751.4	11.0	1.01 (0.96, 1.06)	1.03 (0.99, 1.09)	
≥55	135,176	12,257	1,030,698.4	11.9	**1.10 (1.04, 1.15)**	**1.09 (1.03, 1.14)**	
Reproductive span, y							
< 30	170,474	14,252	1,277,414.9	11.2	1 (ref.)		1 (ref.)
30-34	529,589	44,244	4,043,760.9	10.9	**0.98 (0.96, 1.00)**		1.00 (0.98, 1.02)
35-39	490,384	41,257	3,788,551.8	10.9	**0.98 (0.96, 1.00)**		1.00 (0.99, 1.02)
≥40	81,668	7,142	626,405.9	11.4	1.02 (0.99, 1.05)		1.02 (0.99, 1.05)
Parity							
Nulliparity	32,006	2,817	247,443.9	11.4	1 (ref.)	1 (ref.)	1 (ref.)
1	79,411	6,463	619,475.1	10.4	**0.92 (0.88, 0.96)**	**0.94 (0.89, 0.98)**	**0.93 (0.89, 0.98)**
≥2	1,160,698	97,615	8,869,214.4	11.0	0.97 (0.93, 1.00)	**0.93 (0.89, 0.97)**	**0.93 (0.89, 0.97)**
Duration of breast feeding, mo							
Never	86,477	7,122	673,737.5	10.6	1 (ref.)	1 (ref.)	1 (ref.)
<6	86,290	6,752	675,687.5	10.0	0.95 (0.91, 0.98)	0.97 (0.93, 1.00)	0.97 (0.93, 1.00)
6-11	225,607	18,563	1,750,226.8	10.6	1.00 (0.98, 1.03)	1.00 (0.97, 1.03)	1.01 (0.98, 1.04)
≥12	873,741	74,458	6,636,481.7	11.2	**1.06 (1.04, 1.09)**	1.02 (0.99, 1.05)	1.03 (1.00, 1.06)

© 2021 Yoo JE et al. *JAMA Network Open.*

Duration of HT, y							
Never	1,023,519	86,001	7,801,630.3	11.0	1 (ref.)	1 (ref.)	1 (ref.)
<2	118,146	10,066	922,759.7	10.9	0.99 (0.97, 1.01)	1.02 (1.00, 1.04)	1.02 (1.00, 1.04)
2-4	48,812	4,051	382,048.7	10.6	**0.96 (0.93, 0.99)**	0.98 (0.95, 1.02)	0.98 (0.95, 1.01)
≥5	37,581	2,968	294,392.8	10.1	**0.92 (0.88, 0.95)**	**0.92 (0.88, 0.95)**	**0.91 (0.88, 0.95)**
Unknown	44,057	3,809	335,301.9	11.4	1.03 (1.00, 1.06)	1.03 (0.99, 1.07)	1.03 (0.99, 1.07)
Duration of OC use, y							
Never	1,015,265	84,938	7,762,969.8	10.9	1 (ref.)	1 (ref.)	1 (ref.)
<1	116,384	9,877	898,277.1	11.0	1.01 (0.98, 1.03)	1.01 (0.99, 1.03)	1.01 (0.99, 1.03)
≥1	77,581	6,739	594,891.8	11.3	**1.04 (1.01, 1.06)**	**1.03 (1.00, 1.02)**	**1.03 (1.00, 1.05)**
Unknown	62,885	5,341	479,994.8	11.1	1.02 (0.99, 1.05)	1.01 (0.98, 1.04)	1.01 (0.98, 1.04)

Abbreviations: PYs, person-years; HT, hormone therapy; OC, oral contraceptive
Model 1: crude model
Model 2: the full model included age, age at menarche, age at menopause, parity, duration of breast feeding, duration of HT, duration of OC use, alcohol consumption, smoking, regular exercise, income, body mass index, hypertension, diabetes, dyslipidemia, and cancer.
Model 3: the full model included reproductive span instead of age at menarche and menopause in Model 2.

© 2021 Yoo JE et al. *JAMA Network Open.*

eTable 5. Hazard Ratios and 95% Confidence Intervals of Fractures

Variables	Any fracture	Vertebral fracture	Hip fracture	Other fracture
Income (quartile)				
Q1 (lowest)	1 (ref.)	1 (ref.)	1 (ref.)	1 (ref.)
Q2	1.00 (0.98, 1.01)	1.00 (0.97, 1.02)	1.04 (0.98, 1.11)	0.99 (0.97, 1.01)
Q3	1.01 (0.99, 1.02)	**1.03 (1.01, 1.06)**	1.03 (0.97,1.09)	1.00 (0.98, 1.02)
Q4 (highest)	1.00 (0.98, 1.01)	0.99 (0.97, 1.01)	1.01 (0.96, 1.06)	1.01 (0.99, 1.02)
Smoking status				
Never	1 (ref.)	1 (ref.)	1 (ref.)	1 (ref.)
Ex-smoker	0.97 (0.93, 1.02)	0.93 (0.86, 1.01)	1.16 (0.98, 1.38)	0.94 (0.89, 1.00)
Current smoker	**1.06 (1.03, 1.09)**	**1.13 (1.08, 1.18)**	**1.37 (1.24, 1.52)**	0.98 (0.94, 1.02)
Alcohol consumption				
None	1 (ref.)	1 (ref.)	1 (ref.)	1 (ref.)
Mild	**1.05 (1.04,1.07)**	0.98 (0.96, 1.01)	0.91 (0.84, 0.98)	**1.08 (1.06, 1.10)**
Heavy	**1.26 (1.19, 1.35)**	**1.18 (1.05, 1.32)**	**1.58 (1.19, 2.08)**	**1.26 (1.17, 1.36)**
Regular exercise	**0.98 (0.97, 0.99)**	**0.90 (0.88, 0.92)**	**0.92 (0.87, 0.97)**	**1.05 (1.03, 1.06)**
Body mass index (kg/m^2)				
<18.5	1.02 (0.98, 1.05)	1.02 (0.98, 1.07)	**1.34 (1.21, 1.48)**	**0.94 (0.90, 0.99)**
18.5 to <23	1 (ref.)	1 (ref.)	1 (ref.)	1 (ref.)
23 to <25	1.00 (0.99, 1.01)	**1.04 (1.02, 1.06)**	**0.91 (0.86, 0.95)**	1.00 (0.99, 1.02)
25 to <30	**0.98 (0.97, 0.99)**	**1.06 (1.05, 1.08)**	**0.89 (0.85, 0.93)**	**0.96 (0.94, 0.97)**
≥30	**0.94 (0.92, 0.96)**	**1.08 (1.04, 1.12)**	0.96 (0.87, 1.05)	0.87 (0.85, 0.90)
Comorbidities				
Hypertension	**0.97 (0.96, 0.98)**	**0.96 (0.95, 0.98)**	**1.18 (1.14, 1.23)**	**0.96 (0.95, 0.98)**
Diabetes mellitus	**1.05 (1.03, 1.06)**	**0.86 (0.85, 0.88)**	**1.71 (1.64, 1.79)**	**1.12 (1.10, 1.14)**
Dyslipidemia	**0.98 (0.97, 0.99)**	**0.97 (0.95, 0.98)**	0.99 (0.95,1.03)	1.00 (0.99, 1.02)
Cancer	0.97 (0.95, 1.00)	**0.92 (0.87, 0.96)**	0.97 (0.86, 1.10)	1.02 (0.98, 1.06)

The full model included age, age at menarche, age at menopause, parity, duration of breast feeding, duration of HT, duration of OC use, alcohol consumption, smoking, regular exercise, income, body mass index, hypertension, diabetes, dyslipidemia, and cancer.

© 2021 Yoo JE et al. *JAMA Network Open.*

eFigure. Flow Chart of the Study Population

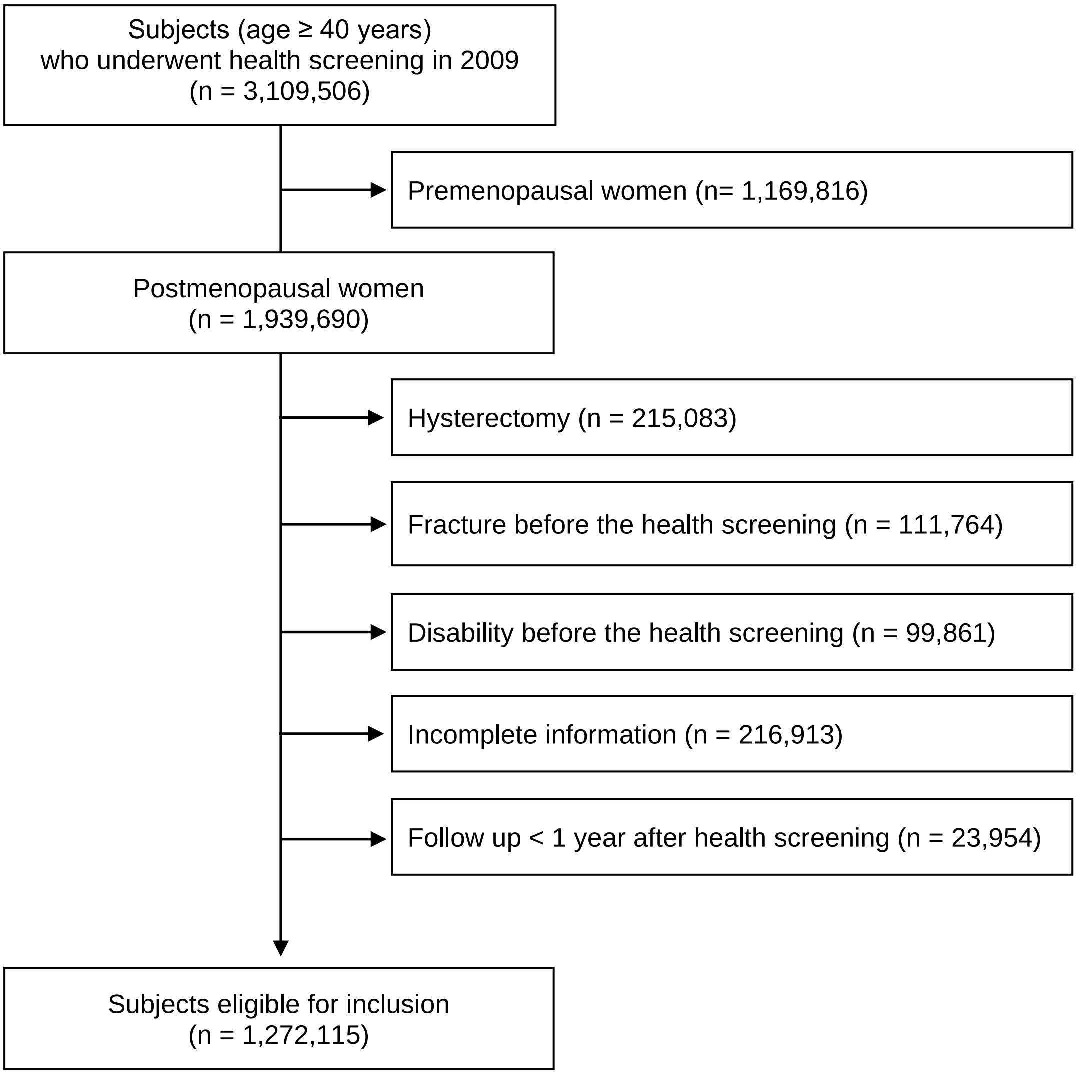

© 2021 Yoo JE et al. *JAMA Network Open.*
